# Supplementary material for: Practical considerations for Ultraviolet-C radiation mediated decontamination of N95 respirator against SARS-CoV-2 virus
Source: PLoS One. 2021 Oct 12;16(10):e0258336. doi: 10.1371/journal.pone.0258336 (PMC8509861; doi:10.1371/journal.pone.0258336)
Supplement: S1 File — (DOCX) [file pone.0258336.s001.docx]

# **S1 File: Extended Material and Methods**

## **Virus preparation and stock titration.**

The SARS-CoV-2 strain used was USA-WA1/2020. Viral stocks were obtained from the Biodefense and Emerging Infections Research Resources Repository. BEI stocks were amplified in Vero-E6 cells (obtained directly from ATCC, ATCC CRL-1586) to produce virus passage 1 and passaged again in Calu-3 cells (obtained directly from the UC Berkeley Cell Culture Facility) to produce virus passage 2 which was used for experiments. For passage 1, 50 ul of the BEI stock was inoculated onto T-175 flasks of Vero-E6 cells and allowed to propagate until 50% cytopathic effect (CPE) was achieved (~48 hours post infection) at which time flasks were lysed through 1 round of freeze and thaw, then supernatants were collected and clarified through a gentle spin step (1500 rpm for 5 mins). The clarified viral supernatants were aliquoted and frozen down at -80C. Aliquots were thawed for titration or production of virus passage 2 which was done the same as above except using Calu-3 human lung epithelial cells. Viral stocks were propagated in Calu-3 cells grown in Dulbecco's Modified Eagle Medium (DMEM) containing 10% FBS and penicillin/streptomycin. The concentration of viral stocks was assessed by TCID_50_ assay using Vero-E6 cells and was determined to be 8 x 10^7^ TCID_50_/ml.

## **UV-C dose measurements**

The UVC LEDs were commercially available products from Bolb, Inc.: Surface Mount Type SMD6060, with peak emission wavelength of 272 nm, full-width-at-half-maximum of 9.5 nm, and outputs of 100 mW (250 mA) or 140 mW (350 mA) per LED. Website: www.bolb.co

The LED emission is narrow, at a wavelength of 272 nm. The range of irradiance incidents upon the surface on 3M 1860 respirators was 1.5 to 3.0 mW/cm^2^. To account for non-uniformity across the surface of a respirator, the irradiance at each inoculation site was measured using a custom 3M 1860 respirator with calibrated sensors (S2 Fig, sensors #84-985 from Edmund Optics). A similar dose validation experiment was performed with a 3M 8210 respirator and showed similar irradiances at each inoculation site (data not shown).
