# Supplementary figures and images for: Practical considerations for Ultraviolet-C radiation mediated decontamination of N95 respirator against SARS-CoV-2 virus
Source: PLoS One. 2021 Oct 12;16(10):e0258336. doi: 10.1371/journal.pone.0258336 (PMC8509861; doi:10.1371/journal.pone.0258336)

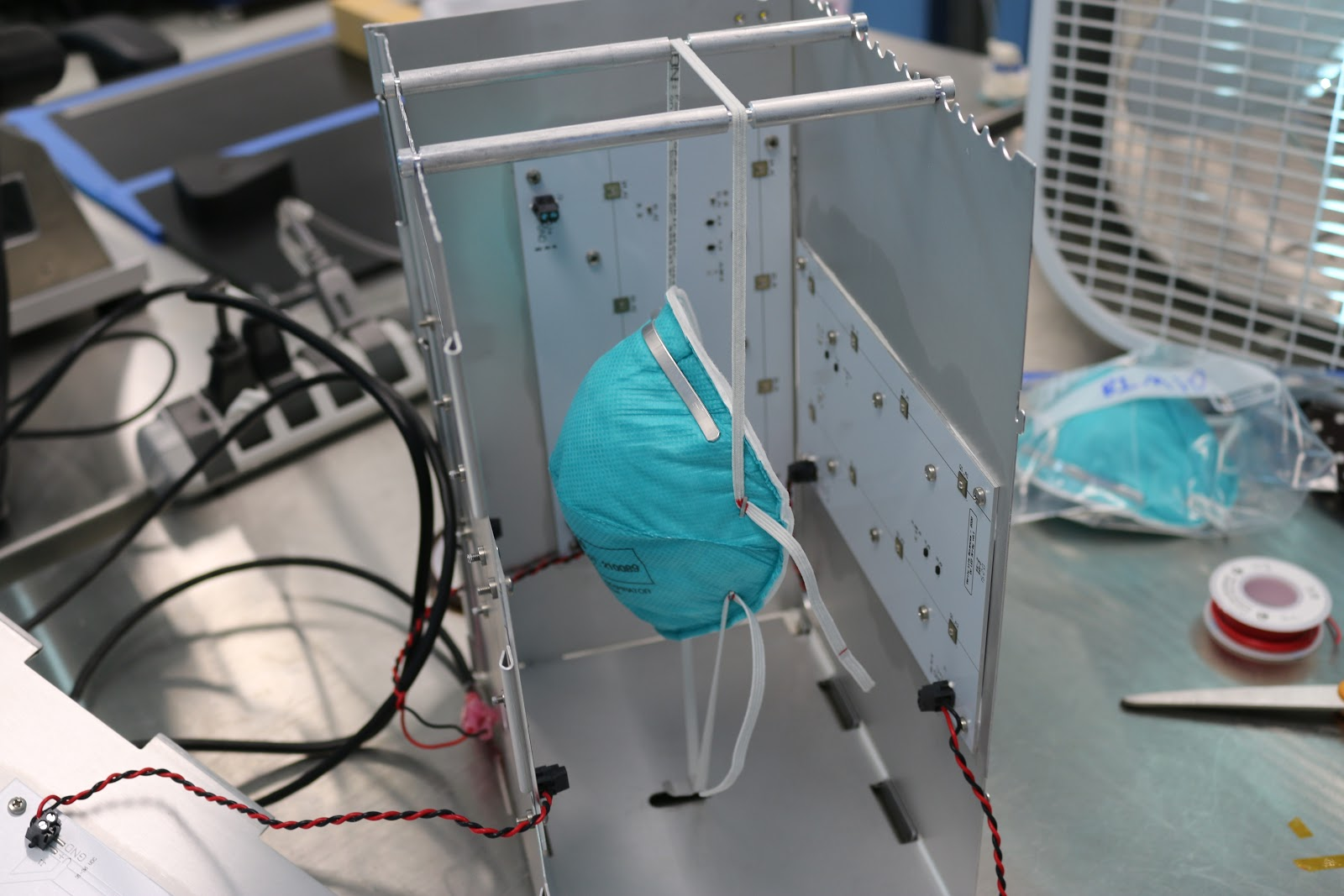

Supplement: S1 Fig — Front panel was removed for visibility. (TIF) [file pone.0258336.s003.tif]

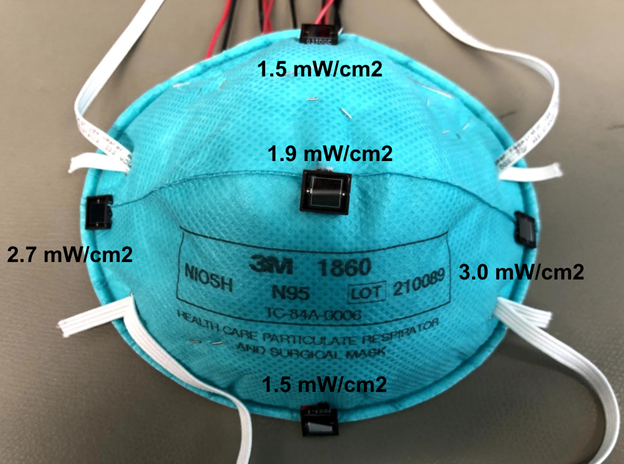

Supplement: S2 Fig — (TIFF) [file pone.0258336.s004.tiff]

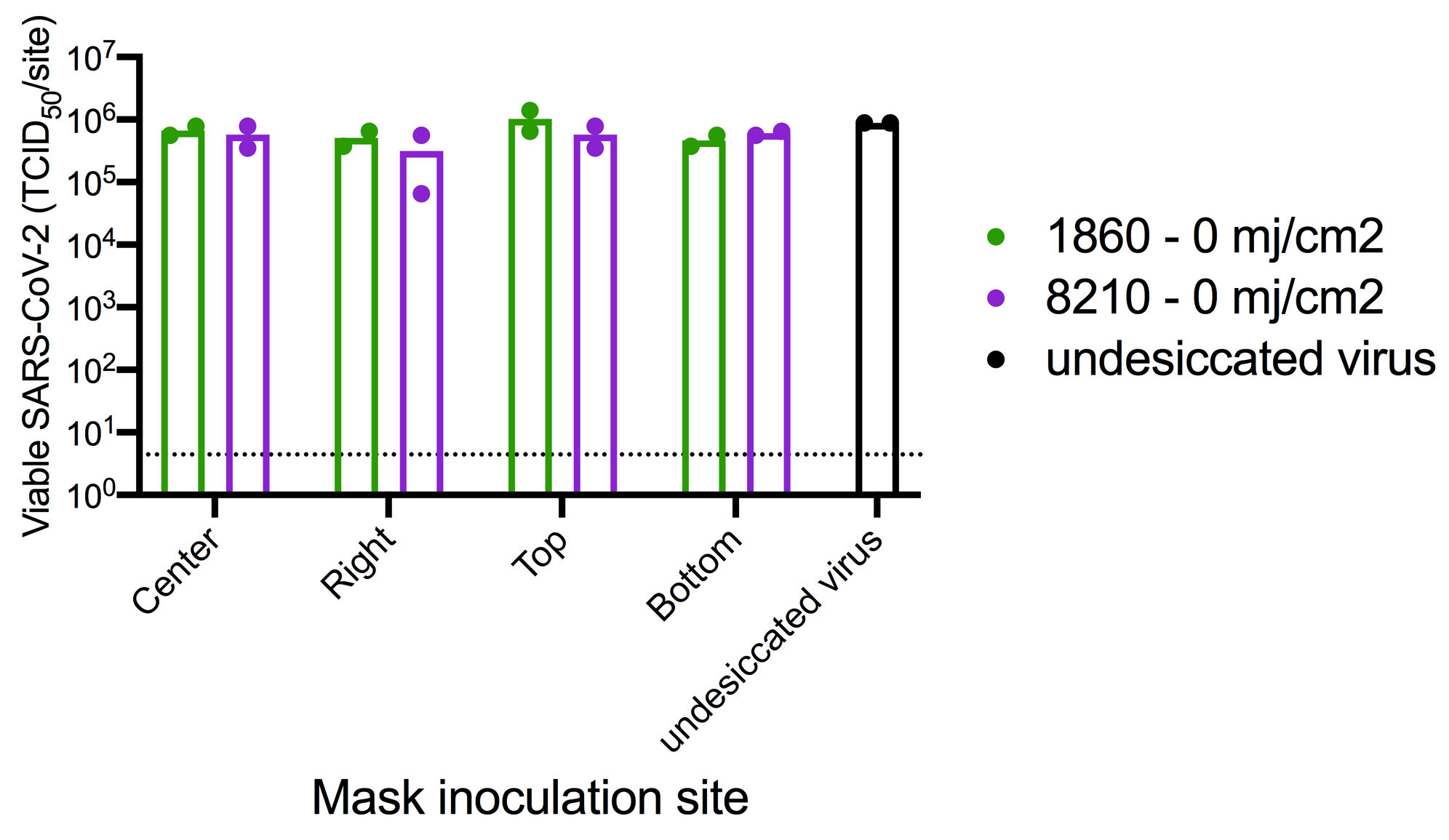

Supplement: S3 Fig — (TIFF) [file pone.0258336.s005.tiff]
