# Supplementary material for: Practical considerations for Ultraviolet-C radiation mediated decontamination of N95 respirator against SARS-CoV-2 virus
Source: PLoS One. 2021 Oct 12;16(10):e0258336. doi: 10.1371/journal.pone.0258336 (PMC8509861; doi:10.1371/journal.pone.0258336)
Supplement: S1 Table — Doses are calculated based on irradiance measurements made with the custom N95 respirator with calibrated sensors. Units are in mJ/cm2. (DOCX) [file pone.0258336.s006.docx]

# **S1 Table: calculated doses delivered at each location for each exposure**

|  | **300 sec** | **600 sec** |
| --- | --- | --- |
| **Right** | 810 | 1620 |
| **Center** | 570 | 1140 |
| **Top** | 450 | 900 |
| **Bottom** | 450 | 900 |
| **Strap** | N/A | N/A |

Doses are calculated based on irradiance measurements made with the custom N95 respirator with calibrated sensors. Units are in mJ/cm^2^.
