# Supplementary material for: Practical considerations for Ultraviolet-C radiation mediated decontamination of N95 respirator against SARS-CoV-2 virus
Source: PLoS One. 2021 Oct 12;16(10):e0258336. doi: 10.1371/journal.pone.0258336 (PMC8509861; doi:10.1371/journal.pone.0258336)
Supplement: S2 Table — (DOCX) [file pone.0258336.s007.docx]

# **S2 Table: maximum log reduction possible at each mask location**

|  | **3M 1860** | **3M 8210** |
| --- | --- | --- |
| **Aluminum** | 4.95 | 4.95 |
| **Right** | 5.06 | 4.85 |
| **Center** | 5.18 | 5.11 |
| **Top** | 5.36 | 5.11 |
| **Bottom** | 5.02 | 5.13 |
| **Strap** | 4.24 | 4.88 |
