# Supplementary material for: Practical considerations for Ultraviolet-C radiation mediated decontamination of N95 respirator against SARS-CoV-2 virus
Source: PLoS One. 2021 Oct 12;16(10):e0258336. doi: 10.1371/journal.pone.0258336 (PMC8509861; doi:10.1371/journal.pone.0258336)
Supplement: Attachment — Submitted filename: Response to reviewers.docx [file pone.0258336.s008.docx]

*Dear Dr. Moore,*

*Thank you for giving us the opportunity to submit a revised draft of the manuscript “Practical considerations for Ultraviolet-C radiation mediated decontamination of N95 respirator against SARS-CoV-2 virus” for publication in PLOS ONE. We thank you for the feedback you and the reviewers gave us on the original manuscript and for the suggestions you made to improve our article. We have incorporated most of the suggestions made by the reviewers. Those changes are highlighted within the manuscript. Please see below, in purple, for a point-by-point response to the reviewers’ comments and questions.*

- the methods section is currently very brief and important information is either missing or included as supplementary material rather than main text.

*We agree that our material and methods section was too brief to permit proper understanding of the methods used in our study. We have moved most of the supplementary material to the method section and have provided more details, particularly on the UV-C device itself.*

- For example, the treatment of control (non-exposed) masks/surfaces should be clarified - was virus recovered after the 3.5 h drying time (0 sec exposure) or were masks placed in the chamber and exposed to 0 mJ/cm2 for 300 and 600 seconds?

*We have clarified the material and method section to explain how non-exposed masks were handled. The masks were not placed in the device but were harvested at the very end of the experiment to control for excessive drying.*

- Similarly, straps were not subjected to the 3.5 h drying time and, whilst it is stated (supplementary data) that no losses in viability occurred over the 3.5h drying time, could desiccation stress increase virus susceptibility to subsequent UV-c exposure?

*We agree that desiccation may increase susceptibility of SARS-CoV-2 to UV-C exposure. We had to let the virus dry on the mask before UV-C exposure because of how the masks are placed in the device (see photo of the device). We have updated the discussion section to mention this point as a limitation of our study.*

- Whilst some of the additional information asked for by the reviewers can be considered supplementary, please ensure sufficient detail is provided within the main text.

*See below for details of the changes.*

- If possible, and as suggested by both reviewers, please also include a picture of the chamber and configuration of the masks within.

*We have added a photo of the device as supplementary material and have updated the material and method section to give a better explanation of how it is operated.*

- I would also draw specific attention to comments made by Reviewer 2 and their concerns relating to data interpretation. Please ensure your conclusions are supported by the data and that limitations of the study have been acknowledged.

*We have incorporated paragraphs to the results and discussion section to acknowledge the following limitations of our study:*

- *Potential impact of desiccation on susceptibility of the virus to UV.*
- *Masks were not tested for fit and filtration.*
- *Only the exterior of the mask was tested for decontamination.*

Reviewer #1: Review of PONE-D-21-00512

- Could do with more results because it was only completed twice for each mask and the results lack some potential significance in interpretation due to it.

*We agree that more masks would have given more statistical significance to the results. However, each mask model was tested in two independent experiments that gave similar results.*

- It would also be interesting to have completed the study on masks that had already been worn because this is how they would be presented when decontaminated in reality.

*This comment correlates with the question by reviewer 2 who asked if the masks were tested for fit and filtration after UV-C exposure. We did not perform such tests in our study. We have added a sentence in the discussion section to acknowledge this point as a limitation.*

- There is no evaluation of the effects angular incidence causes on the decontamination results as mentioned in the introduction.

*We have corrected the introduction to mention only the 3D structure of the mask.*

Specific questions:

- Why did you choose the sites for the contamination of the masks?

*The following sentence was added to the method section to explain how the sites were selected: “The locations were selected after consideration of the 3D structure of the mask and corresponding differences in irradiation dose received to cover a wide range of anticipated doses”.*

- Why did you only test two models of the mask? Was it because these were the only ones being used in the University or were they chosen because their properties were thought to give different results from the exposure?

*We have added a paragraph to the Material and Method section to discuss why we selected the two mask models for the study. Briefly, many mask models were incompatible with UV-C decontamination (masks with valves or folding masks). Both mask model used in our study are approved for healthcare worker use during the COVID-19 pandemic, but they present different properties (shape, material) that we thought were interesting to test.*

- The last sentence in the introduction is not needed.

*We removed the sentence.*

- How did you stop the droplet inoculum from spreading on the non-horizontal surfaces if they were all inoculated at the same time? How does this vary from just adding 50ul in one go? Were the 3 inoculums added to exactly the same spot? Did you add each inoculum and leave it to dry prior to adding the next? If not added together how does this represent contamination when masks are used in practice?

*We have updated the material and method section to give a more thorough explanation of how the virus was inoculated on the masks. Preliminary experiments showed that simultaneous addition of 3 adjacent droplets was the optimal method of inoculation to reduce drying time and prevent droplets from falling from the mask.*

- More information on the UVc exposure chamber is needed and positioning of the masks inside it, or was each mask exposed separately? Maybe a picture of the chamber would help in the description.

*We have expanded the material and method section and added a picture of the device. The chamber can hold only one mask at a time.*

- the brief section in the results/discussion section should be moved into the materials and methods.

*The paragraph was moved to the material and methods.*

- Was there a temperature increase in the exposure device during operation?

*A new sentence was added to the material and method section to specify that temperature remained below 41 deg C for all disinfection runs.*

- How was the CPE visualized, was a stain used?

*The following sentence was added to the material and method section: “cytopathic effect, defined as any virus-induced cell death or change in cell morphology, was scored visually under brightfield illumination using a 4X / 0.13 NA objective.”*

- Please explain what were the aluminum coupons used for (virus titration section)? Why were aluminum coupons chosen over stainless steel for coupons?

*Aluminum coupons were used as a smooth and non-porous control surface that could be attached directly to the mask during UV-C exposure. Aluminum coupons were selected over stainless steel because they could be easily bent to follow the shape of the mask without causing shadowing effects. We have incorporated this explanation to the material and method section.*

- Were the coupons excised from the mask agitated in the recovery medium?

*The following sentence was added for clarification: “Samples were incubated in […] DMEM […] for a minimum of 30 minutes and subjected to gentle manual agitation at the beginning and end of the incubation.”*

- What was the loss of viability over the course of the experiment from exposure to the environmental conditions inside the UVc chamber without the LEDs being switched on?

*This parameter was not tested, although the time spent inside the chamber was very short (<15 min) compared to the drying time (3.5 h) and therefore unlikely to cause a significant loss of viability. Non-irradiated control masks were treated last to account for this parameter.*

- Could an increase in heat have contributed to the reduction in viability of the virus?

*We have added the following sentence to the material and method section to address this point: “The temperature of the metal core circuit board, to which the UV LEDs were mounted to, remained below 41°C for all disinfection runs.”*

- Please include a reference for the statement identifying that a 3-log reduction is the industry standard for an effective decontamination.

*We followed recommendation made by Reviewer 2 to cite the* *FDA’s “recommendations for sponsors requesting EUA’s for decontamination and bioburden reduction systems for surgical masks and respirators during the (COVID-19) public health emergency” document.*

- The results from the aluminium show a close to 5 log reduction, not an actual 5 log reduction. The text needs to be changed to reflect this.

*We have corrected the text.*

- How were the aluminium coupons presented in the chamber and how does this relate to the inoculated masks (distance, orientation)?

*The aluminum coupons were taped to the center of the irradiated masks. They were precisely positioned to receive irradiation doses equivalent to the “center” mask location. We have added a sentence to Method section to clarify this point.*

- What was the maximum log reduction that could be achieved in this assay?

*The maximal log reduction in our assay is approx. 5 logs. We have added a supplementary file recapitulating the maximum log reduction at each mask location.*

- Some more interpretation of the surface types would be good for the discussion. You mention the 1860 was hydrophobic, but being made from polyester, was the 8210 hydrophobic as well? How did the inoculum present on the surfaces i.e. did they stay as droplets on the material or were they absorbed, and how do you think this would have affected the exposure?

*We observed similar drying time for both mask models, although the texture of the outer layer was very different. Interestingly, the 8210 masks present a rougher outer surface. We hypothesize that it could perhaps shield the virus from some of the UV-C exposure. We have modified the results and discussion section to discuss this point.*

- Do you think the colour differences of the masks played a role in the decontamination?

*This is an interesting question, but we have no mean to test it.*

- How close were your exposure doses to those that would compromise material integrity?

*We have added the following sentence to the discussion to address this point: “Importantly, the UV-C doses used in our study were considerably lower than the cumulative doses reported to degrade N95 material (Lindsley et al. J Occup Environ Hyg 2015)”.*

- You mention that “Details on virus propagation can be found in supplementary materials” but that is in the supplementary file. Please clarify.

*We removed the sentence.*

- What type of Biosafety cabinet was used for the drying of the masks?

*We have added the brand of the biosafety cabinet to the material and method section (**Nuaire LabGard model NU-540-600).*

Reviewer #2:

- would benefit from a more comprehensive review of the literature in the introduction and discussion.

*We have developed the introduction section to give a more comprehensive review of the literature. Notably, we have included more references to older studies done with viruses other than SARS-CoV-2.*

- The article could be improved upon by including a more in depth look at current respirator UV-C disinfection practices and the advantages / disadvantages of these methods.

*UV-C decontamination can be performed in a wide variety of systems in which many variables come into play (type, number and position of light sources, number of masks that can fit into the device, positioning of the mask into device etc.) We think that discussing these variables would make an excellent topic for a review but is beyond the scope of this manuscript.*

Questions and comments:

- Page 2. In the introduction, address if any facilities are using UV-C decontamination for respirators currently or during the peak of the pandemic? This would put the article into context with current practices and would be helpful either in the introduction or discussion. Examples are Ozog et al. 2020 and Golladay et al. 2021.

*We have added a paragraph to the introduction to reference the two examples cited.*

- Page 2: In the introduction or discussion include the benefits / disadvantages to respirator disinfection with UV-C vs. other methods.

*We have updated the introduction to mention the two other most promising methods of N95 decontamination and we have added references to an excellent review discussing the benefits and disadvantages of each method (Torres et al.).*

- Page 3. Instead of referring to the ambiguous industry standard it would be better to cite the FDA’s “recommendations for sponsors requesting EUA’s for decontamination and bioburden reduction systems for surgical masks and respirators during the (COVID-19) public health emergency” document.

*We thank reviewer 2 for this suggestion. We have modified the manuscript as recommended.*

- Include in the discussion or introduction that there is currently only 1 UV-C device with an EUA for respirator disinfection.

*This is now mentioned in the introduction.*

- Page 4. Mask inoculation and UV-C exposure – indicate that only the exterior of the mask was tested. This limits use of the respirator to the to the same user. Do you have a comment on importance (or lack there of?) of disinfecting the internal fibers and interior of the respirator?

*We have added a paragraph to the discussion section regarding this point. The FDA states that the use of respirators by single-users poses less risk of unintended transmission of SARS-CoV-2 or other pathogens. Limiting mask decontamination to single users may be a safer option during times of shortages.*

- Page 4. The description of the UV-C disinfection device should be under materials and methods, not results and discussion heading.

*We have moved this paragraph to the material and method section.*

- If possible, it would be helpful to include an image of the device and placement of the mask in the supplementary files.

*We have expanded the material and method section and added a picture of the device.*

- S1 File: Extended Material and Methods page 2 – indicate source and model for calibrated UV-C sensors.

*This information has been added to the S1 File.*

- Page 5. Figure: UV-C decontamination of multiple locations on two models of N95 respirators: Panel B) Indicate in the legend that it is showing each site independently with the dots then an average of all sites with the bar graph.

*We have modified the Figure Legend accordingly.*

- Page 5. Reference FDA recommendations instead of “industry standard”.

*We have updated the text as suggested.*

- Page 7 line 3 – Were irradiation doses carried out on the 8210 model as well? If the irradiation doses on the 8210 and 1860 are the same in the various locations, then the material of the mask is the major influencing factor. It is possible that there is a small change in the shape of the 8210 compared to the 1860 that hinders the efficacy of UV-C.

*The validation of the irradiation doses was carried out on both mask models. We observed similar irradiance levels for the two mask models. We have clarified this point in the method section.*

- Page 7 line 14 and 15 – if you conducted this same testing on swatches of mask would you see this same result? This would solidify that it is the material that is the major factor in efficacy.

*Unfortunately, we could not test mask swatches because the device we used for the study was designed to fit whole masks (see photo). We agree that we do not bring definitive proof that mask material is the major factor contributor to UV-C efficacy, and we have toned down our conclusions accordingly.*

- Did treated masks pass fit and filtration testing? If not tested, it needs to be indicated as a limitation of the study.

*We have added a paragraph to the discussion section to acknowledge this point as a limitation of the study.*

- Testing of only the exterior of the mask needs to be included as a limitation of the study.

*This point is now mentioned as a limitation of the study in the discussion section.*

- Discussion: It would make sense that the center of the mask would have the highest irradiation levels since it is closes and most parallel to the LEDs however it does not. The best reduction is occurring at the edges of the mask, any thoughts on why that is?

*This is due to the design of our UVGI device. Unlike most UVGI devices that have only one light source, our UVGI device has LEDs mounted on every vertical wall. When the mask is in the device, the edges of the mask are closer to the light source than the center of the mask and receive higher irradiance (See Fig S1), which result in a greater reduction in viral titer.*
